# Supplementary material for: Exploring the Impact of Extra Virgin Olive Oil on Maternal Immune System and Breast Milk Composition in Rats
Source: Nutrients. 2024 Jun 6;16(11):1785. doi: 10.3390/nu16111785 (PMC11174597; doi:10.3390/nu16111785)
Supplement: Supplementary file 1 [file nutrients-16-01785-s001.zip › nutrients-3036068-supplementary.pdf]

Supplementary Materials

Table S1. Primers used to carry out the PCR quantitative assay.

	**Gene**	**Cat# Reference**
Occludin	*Ocln*	Rn00580064_m1
ZO-1	*Tjp1*	Rn02116071_s1
Claudin-4	*Cldn4*	Rn01196224_s1
MUC-2	*Muc2*	Rn01498206_m1
MUC-3	*Muc3*	Rn01481134_m1
TLR2	*Tlr2*	Rn02133647_s1
TLR4	*Tlr4*	Rn00569848_m1
TLR5	*Tlr5*	Rn04219239_s1
TLR7	*Tlr7*	Rn01771083_s1
TLR9	*Tlr9*	Rn01640054_m1
IgA	*IgA*	331941, made to order
GPR43	*Ffar2*	Rn02345824_s1

Table S2. Growth-associated variables and relative organ weight at L21.

**Growth variables**	**REF**	**ROO**	**EVOO**
Body length (cm)	21.00 ± 0.30	20.70 ± 1.30	20.63 ± 0.15
Body + tail length (cm)	38.05 ± 0.39	37.25 ± 1.45	37.60 ± 0.42
Body/tail length ratio	0.55 ± 0.01	0.56 ± 0.01	0.55 ± 0.00
BMI (g/cm^2)	0.55 ± 0.01	0.51 ± 0.04	0.54 ± 0.01
Lee Index	296.16 ± 4.07	291.88 ± 14.27	297.36 ± 1.94

**Organs weight**	**REF**	**ROO**	**EVOO**
Brain (%)	0.82 ± 0.01	0.85 ± 0.02	0.85 ± 0.02
Heart (%)	0.37 ± 0.02	0.39 ± 0.01	0.39 ± 0.01
Liver (%)	4.34 ± 0.20	4.44 ± 0.12	4.31 ± 0.09
Right kidney (%)	0.38 ± 0.02	0.36 ± 0.02	0.39 ± 0.01
Salivary gland (%)	0.09 ± 0.00	0.08 ± 0.00	0.09 ± 0.00
Spleen (%)	0.17 ± 0.01	0.15 ± 0.01	0.17 ± 0.01
Thymus (%)	0.16 ± 0.01	0.13 ± 0.00	0.15 ± 0.01

BMI, Body mass index; REF, reference group; ROO, reference olive oil group; EVOO, extra virgin olive oil group. Organ weights expressed in g of tissue/ 100 g of body weight and growth variables are expressed as mean ± S.E.M (n=4-6).

Table S3. Hemogram of blood at the end of the study (L21) in reference group (REF), reference olive oil group (ROO), and extra virgin olive oil group (EVOO).

	REF	ROO	EVOO
Leukocytes (x10^9/L)	3.97 ± 0.94	3.50 ± 0.98	3.02 ± 0.50
Lymphocytes (x10^9/L)	1.57 ± 0.37	2.20 ± 0.83	1.32 ± 0.08
Monocytes (x10^9/L)	0.20 ± 0.06	0.13 ± 0.03	0.16 ± 0.06
Granulocytes (x10^9/L)	2.20 ± 0.78	1.67 ± 0.12	1.54 ± 0.42
Lymphocytes (%)	42.67 ± 8.23	42.67 ± 8.23	61.60 ± 6.29
Monocytes (%)	6.53 ± 0.46	6.94 ± 0.78	4.73 ± 0.22
Granulocytes (%)	50.80 ± 8.63	44.12 ± 5.02	33.67 ± 6.23
Platelets (x10^9/L)	574 ± 65.77	473.67 ±71.30	435 ± 84.38
Erythrocytes (x10^9/L)	7.64 ± 0.46	8.60 ± 0.37	7.53 ± 0.49
HGB (g/L)	141.00 ± 3.46	146.00 ± 5.51	132.80 ± 9.56
HCT (%)	38.90 ± 2.11	44.17 ± 1.90 *	38.10 ± 2.44 #
VCM (fL)	51.00 ± 0.60	51.43 ± 0.37	50.65 ± 0.34
HCM(pg)	18.47 ± 0.78	16.93 ± 0.12	17.54 ± 0.36

Results are expressed as mean ± S.E.M (n=4-6). *$p<0.05$ vs REF, #$p<0.05$ vs ROO.

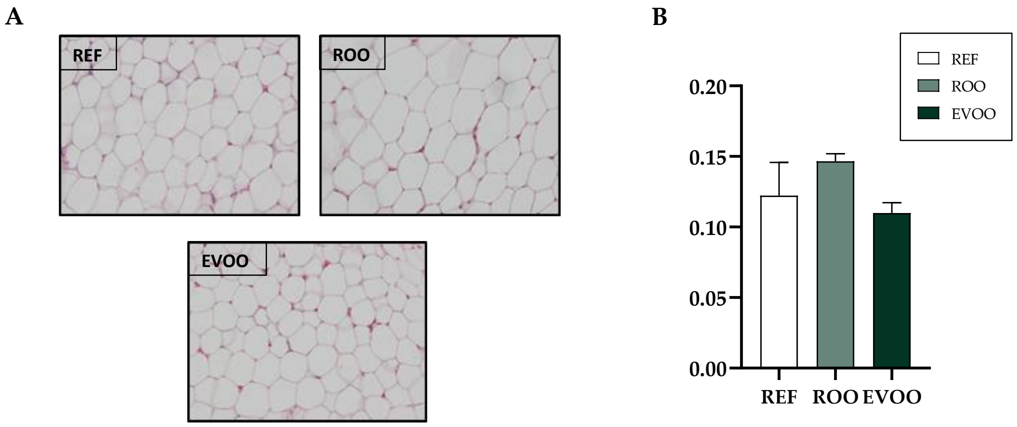

Figure S1. Effect of oils on parametric adipose tissue. (**A**) Histology of parametric adipose tissue. (**B**) Adipocyte area/number of adipocytes. Results are expressed as mean ± S.E.M (n = 4-6).

Briefly, parametric white adipose tissue (WAT) from each animal was selected for histomorphome-try by immersing it in 4 % buffered formaldehyde solution for 24 h at room temperature. Then, the samples were rinsed in a phosphate-buffered solution (PBS) until dehydration in graded ethanol solutions (70 %, 90 %, and 100 %), permeated in xylene and finally embedded in melted paraffin. Paraffin sections (5 µm) were stained using hematoxylin-eosin (HE). Olympus BX41 and Camera Olympus XC50, Olympus (Barcelona, Spain) was used to examine the samples. For each sample of white adipose tissue (WAT) (20x), representative photos were taken. All histology samples were analyzed using Image J (Image Processing and Analysis in Java, National Institute of Mental Health, Bethesda, MD, USA).

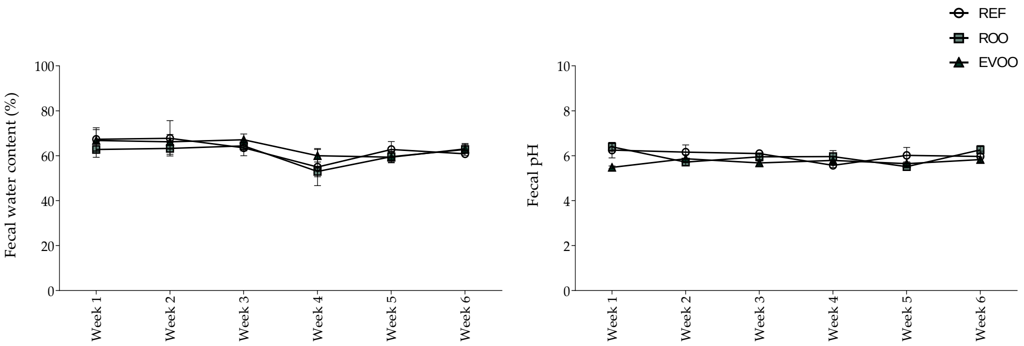

Figure S2. Time course of fecal water content and pH during the study. Results are expressed as mean ± S.E.M (n=4-6).

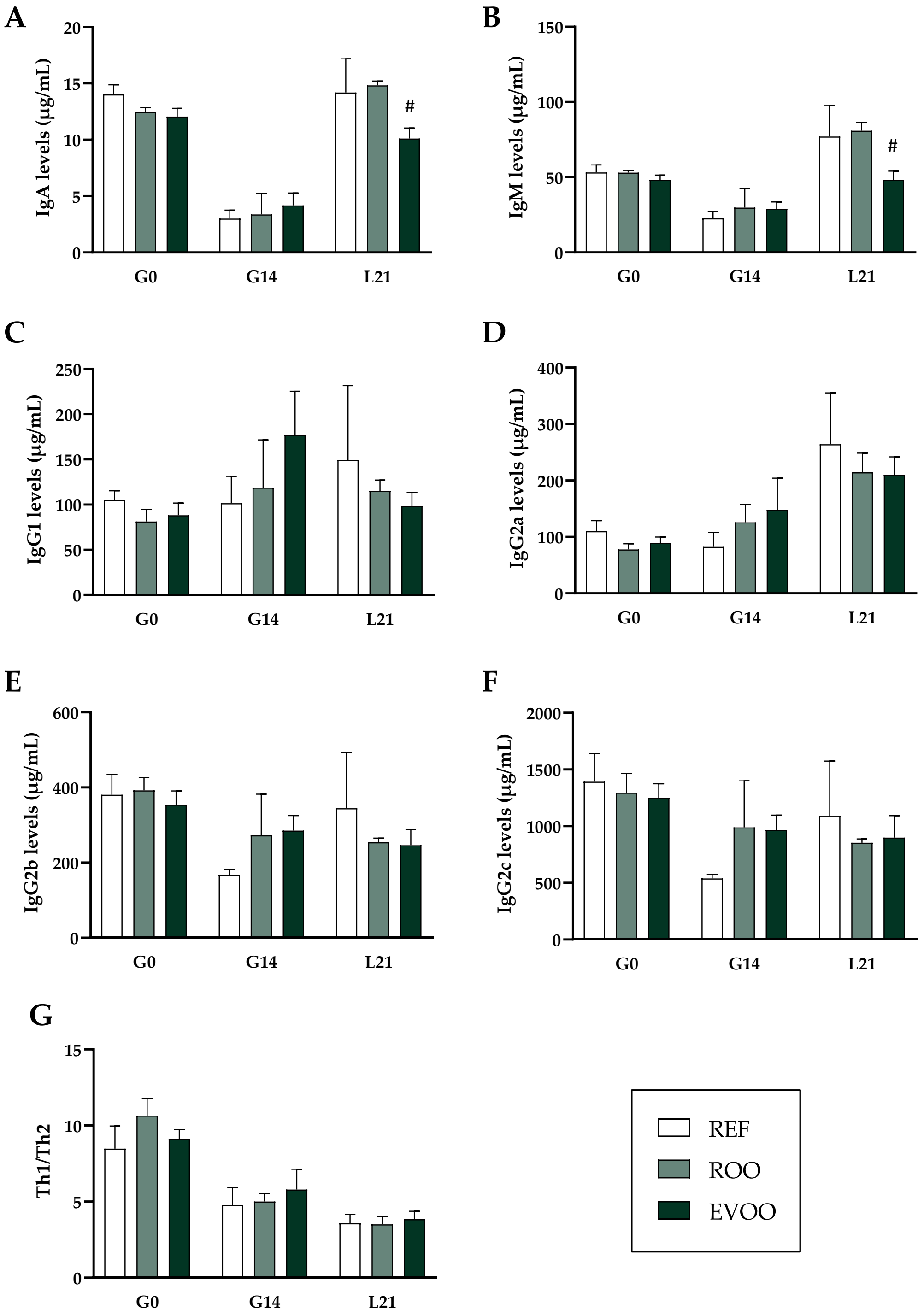

Figure S3. Plasma Ig concentrations on plasma before gestation (G0) at day 14 of gestation (G14) and at the end of lactation (L21). Th1/Th2 = (IgG2b+IgG2c) / (IgG1+IgG2a). Results are expressed as mean ± S.E.M (n=4-6). # $p<0.05$ ROO vs EVOO.

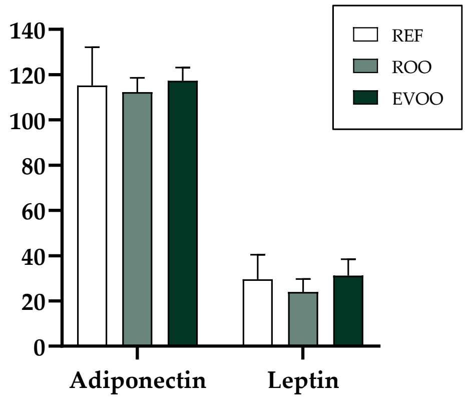

Figure S4. Concentration of adiponectin and leptin in plasma. Results are expressed as mean ± S.E.M (n=4-6). Levels were quantified following ELISA kits manufacturer's instructions (Abcam, Cambridge, UK)

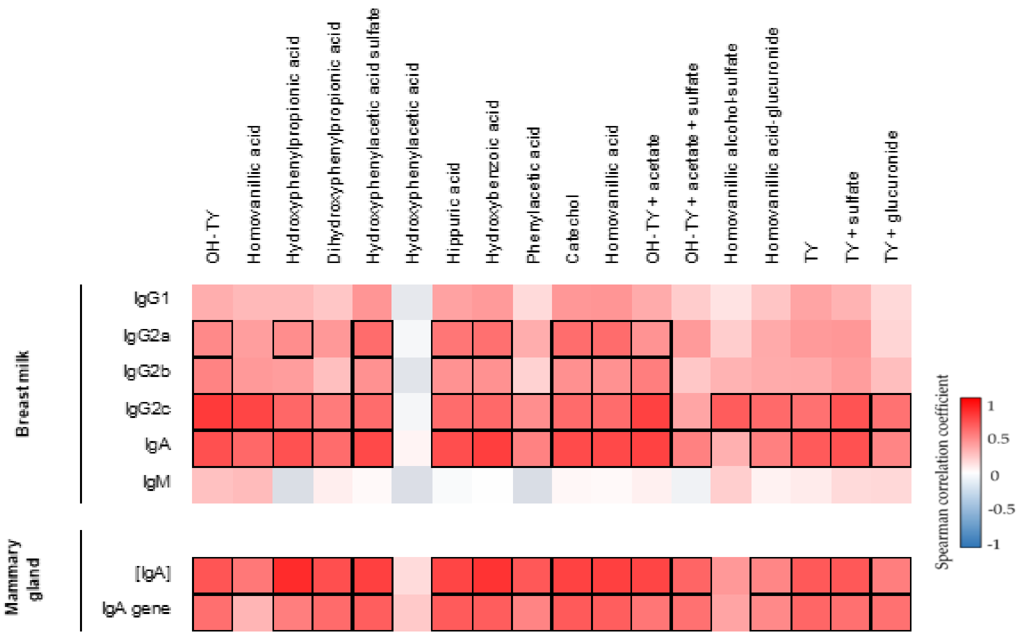

Figure S5. Spearman correlations between breast milk EVOO metabolites and Igs in breast milk and mammary gland. The spearman correlation coefficient is represented in the heat map following the color in the legend. Bold frames represent correlations with statistical significance (p<0.05).
